# The role of telepathology in improving cancer diagnostic and research capacity in sub-Saharan Africa

**DOI:** 10.3389/fmed.2022.978245

**Published:** 2022-10-17

**Authors:** Dana Razzano, Kaushik Puranam, Tamiwe Tomoka, Yuri Fedoriw

**Affiliations:** ^1^Department of Pathology, Stanford University, Stanford, CA, United States; ^2^School of Medicine, Georgetown University, Washington, DC, United States; ^3^Department of Pathology, UNC Project Malawi Cancer Program, Lilongwe, Malawi; ^4^Department of Pathology, University of North Carolina at Chapel Hill, Chapel Hill, NC, United States

**Keywords:** telepathology, cancer, low- to middle-income countries (LMICs), pathology, Africa

## Abstract

Non-communicable disease (NCD), including cancer, disproportionately affect Low- and Middle-Income Countries (LMICs). This inequity is in part due to limitations of pathology services, both human and infrastructural. While significant improvements have been made to address these gaps, creative approaches that are mindful of regional priorities, cultural differences, and unique local challenges are needed. In this perspective, we will describe the implementation of telepathology services in sub-Saharan Africa (SSA) that serve as cornerstones for direct patient care, multi-disciplinary care coordination, research programs, and building human capacity through training. Models and challenges of system implementation, sustainability, and pathologist engagement will be discussed. Using disease and site-specific examples, we will suggest metrics for quality control and improvement initiatives that are critical for providing high-quality cancer registry data and necessary for future implementation of therapeutic and interventional clinical trials.

## Introduction

The impact of non-communicable diseases (NCDs), particularly cancer, represents a major health crisis in low- to middle-income countries (LMICs) that constitute the entirety of sub-Saharan Africa (SSA). In 2020, ~60% of new cancer diagnoses occurred in LMICs (1), where only 5% of global spending is directed toward cancer control (2). Moreover, cancer-related deaths outnumber those related to HIV/AIDS, Tuberculosis, and Malaria (3) and by 2030, 75% of global cancer deaths will occur in LMICs (4). Social and financial consequences of the disproportionate premature deaths in LMICs pose a significant obstacle to social and economic growth and equity. While the reasons for these disparities are multifactorial, lack of timely and accurate tissue diagnosis represents a critical defect in effective systems of cancer care. Tissue diagnosis is necessary for the provision of cancer services to the individual patient but is also critical for the development of comprehensive national/regional cancer registries and clinical trial implementation, that in turn, inform and guide public policy.

These far-reaching and population-level impacts of effective pathology services include development of cancer registries, disease surveillance programs, and national preventative care strategies. In this perspective, we focus on the gaps in pathology services in SSA, progress in diagnostics largely driven by responses to communicable disease burden, and the development of pathology and telepathology programs that aim to improve cancer care in the region.

### Human and infrastructural limitations to effective pathology services in SSA

Recent data from the Lancet Commission on global diagnostics has found that only 47% of the global population has access to laboratory medicine services (5). The critical need for pathology services and gaps to effective deployment of pathologists and equipment in SSA have been extensively described over the past decade (6–14). Survey data published in 2012 (11, 14) demonstrated that with the exceptions of Botswana and South Africa, all countries in SSA have fewer than one pathologist for every 500,000 persons. Many countries have one per million people, and Somalia at that time had no pathologists. For reference, in 2010 the US had an estimated one clinical or anatomical pathologist for approximately every 20,000 persons. The lack of laboratory facilities, reagents and other supplies, advanced technology, infrastructure, supportive staff, and trained laboratory technicians further widens the gap (15). The education of physicians stalls behind more developed countries, leaving a doctor shortage that is not easily compensated. One estimate anticipates that it would take over 400 years to train the number of pathologists needed to have an adequate diagnostic workforce in the current educational system (11).

With respect to human capacity, the divide between the relative resource-rich and resource-poor countries within the region is not necessarily improved by current training paradigms. Physicians who must leave their country of origin to complete pathology programs in high-resourced settings or in high income countries (HICs), find themselves in a challenging situation after training. With newly gained skill and developing expertise in state-of-the art diagnostics, returning to their home country to practice under significant limitations in pathology capacity beyond basic histology and without access to ongoing educational activities for growth and development is challenging. This “brain-drain” has been documented for physicians leaving SSA as a whole (16–19), but also occurs *within* the region with many health workers moving from the public to the private sector, or from rural to more developed urban areas. Furthermore, those who do return are often promoted to leadership and administrative positions that limit time and effort to devote to the clinical practice of pathology or to developing cancer research programs. In addition, they are more likely to suffer from increased stress and burnout (20).

Infrastructural limitations inhibiting ability to provide timely and accurate diagnoses consists of many challenges including lack of physical laboratories with sufficient equipment, reagents, and capacity to process patient samples, among other things (21). Availability of diagnostic tests varies between LMICs and is relative to how many primary care vs. advanced care facilities are available (13). Major gaps in availability are most pronounced in the local primary care settings.

To help address these shortages, the WHO created a list of priority medical devices for cancer management in 2017 (3) with the goal of increasing access especially in LMICs. Pathology and laboratory medicine services are covered under a separate section and consists of an exhaustive list of instruments, reagents, and personnel supply that are the basic minimum needs for providing cancer care. When broken down to testing needs by tier of laboratory, the costs range from modest to enormous from the primary setting to the national/regional referral center respectively (21). Coupled with the essential treatment lists, a clear plan to cover basic need has been created (22). However, a lack of population disease data and continued underinvestment of pathology services in LMICs makes exact quantification of costs to meet the need difficult.

### Interventions and the importance of leap frogging

Interventions must be multifaceted and collaborative to face this complex challenge. The answer is not going to be as simple as training more pathologists in the current educational system in LMICs for instance. Insufficient educational infrastructure currently exists to train an adequate number of pathologists and highly skilled laboratorians using the current systems in place; so it is obvious that advanced education and access to trained pathologists outside of LMICs must occur concurrently for delivering equitable patient care to meet the immediate and future demand.

“Leap Frogging” is a concept that is often applied to global health and essentially means to find a solution to a problem that avoids previously unavoidable steps. A common example in pathology is the use of point of care (POC) testing, such as the Cepheid's Xpert MTB-RIF test that obviates the need for traditional reagents, instruments, laboratory facilities, highly trained technologists and so forth to deliver a rapid diagnosis of drug resistant tuberculosis (8). The medical community must continue to focus on technological advances that leap frog traditional methods of providing care and look for solutions that avoid historical obstacles altogether.

### Role of telepathology

The role of *telepathology*, broadly defined as electronic “sharing” of histopathologic images and clinical data, has evolved in parallel with improvements in technology and communication, and expanded with lower production costs, philanthropic efforts, large-scale public-private partnerships that focus on closing the gap in low-resource settings, and collaborative research programs. Four types of telepathology platforms have been defined and include: (1) static imaging, (2) whole-slide scanning, (3) dynamic nonrobotic telemicroscopy, and (4) dynamic robotic telemicroscopy, and their selection is dependent on local need and/or available resources (23, 24). Unsurprisingly, considerations for platform selection and implementation include technical and resource constrains, including consistent power supply, reliable internet, access to supporting hardware (i.e., computers and servers), and trained staff. However, each of these formats has been used effectively in SSA, and some of these programs have been well-described in published literature (25–50).

**Figure 1:**
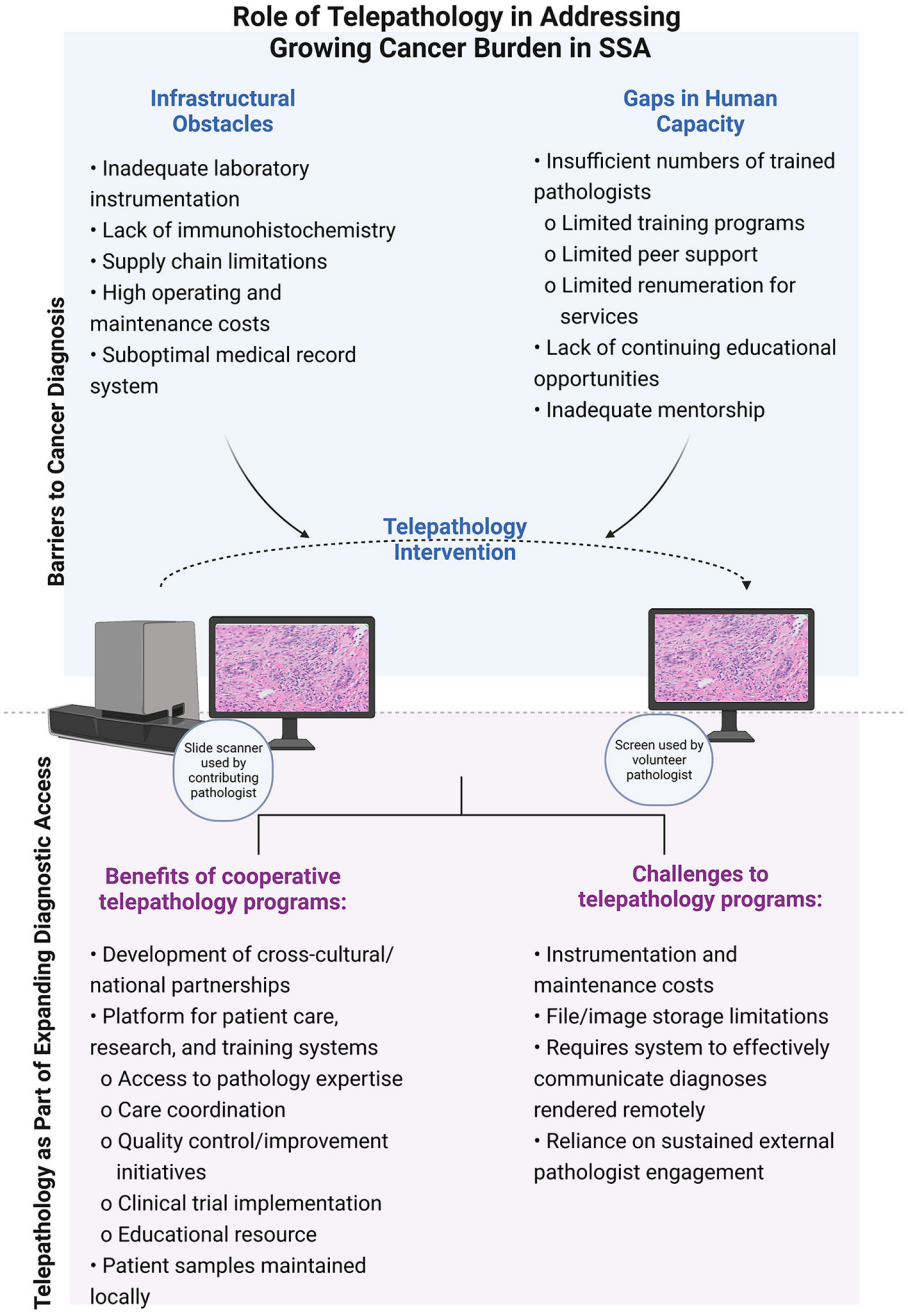
The role of telepathology in addressing the growing cancer burden in sub-Saharan Africa.

Ultimately, deployment of telepathology for cancer care in SSA depends on long-distance engagement of trained pathologists and a sufficient source of funding for implementation. These programs exist on the spectrum from those primarily supported by organizations with international collaboration priorities funded by member and sponsor support to structured research programs funded through grants. Although it has been an excellent example of leap frogging access to care, it is by no means the catholicon solution. In brief, slides still have to be made which requires infrastructural and human capacity as discussed above. As they are rarely revenue-generating, sustainability of telepathology-based intervention programs relies on local, regional, and national governmental, as well as continued international support. A summary of the challenges and barriers facing pathologists in LMICs as well as the benefits and challenges of deploying telepathology in these settings is highlighted in Figure 1. Irrespective of their path to development and deployment, telepathology-based initiatives have significantly changed the face of clinical cancer care, pathology training, and cancer research in SSA. Below, we describe how use of telepathology has paved the way for advances in cancer care, improving pathology training, and expanding research access equity.

#### Clinical cancer care and pathology training

With the revolution of digital pathology, numerous telepathology solutions (51) for providing patient care have evolved to include solutions for global pathology delivery. Some of these projects have been initiatives started by large pathology organizations such as the ASCP/DUKE/UCSF/MOTIC (52) partnership that has successfully installed MOTIC slide scanners in multiple laboratories throughout Africa. The pathologists on site are then able to consult with volunteer pathologists around the United States for second opinions and/or aid with diagnoses. This has also served as a method of supplemental education for the pathologists in low resource settings. This is a major step forward since the pathologists working in LMICs usually lack access to continuing medical education in their daily practice. Another organization that has linked physicians working in LMICs with expert volunteers around the world is Project Echo (53).

Project Echo uses digital pathology and static images to hold multidisciplinary tumor boards that incorporate trainees and mentoring opportunities as a way to educate and support colleagues in low resource settings. They now have 146 programs in 13 different countries and have reported amazing success.

Other groups and individuals have harnessed the power of the internet to deliver high quality didactics aimed at pathologists who have limited access to advanced training opportunities and continuing medical education. Dr. Jerad Garner is a US based soft tissue and dermatopathologist who creates educational content on YouTube and reports frequent views from pathologists in LMICs. For years, he has also led and participated in Facebook discussion groups (Facebook, Inc., Menlo Park, California) to educate patients with rare diagnoses.

Dr. Kamaljeet Singh is a US based breast pathologist that has worked with learners in various sites across the US and internationally to facilitate case-based learning while in-person opportunities were stunted by the COVID pandemic. He and his team used Google Classroom (Google, LLC, Mountain View, California) technology to accomplish this and reported positive feedback from the learners in the program (54).

In response to stifled educational opportunities for medical students to gain practical access to pathology rotations during the COVID pandemic, Dr. Kamran Mirza and Cullen Lilley created a virtual medical student shadowing experience. Participants can create a free account on www.PathElective.com and virtually participate in pathologist-led courses that were curated specifically for medical students, and they even have the opportunity to earn certificates of completion.

A newly formed group of pathologists from around the world have collaborated online to form a new organization, called Open Pathology Education Network (OPEN) aimed at educating pathology residents in low-resource settings. The group will focus its efforts on supporting colleagues in LMICs by providing access to free high-level training using digital pathology, the internet, and video-conferencing among other virtual tools.

It is worth noting that prior to the internet, there were pathologists working to support colleagues in low-resource settings such as Dr. David Kaminsky who started “Africa Calls,” a telephone-based case consultation service. These are just a few examples of some of the ways that organizations and pathologists around the world have collaborated to improve patient care.

Other methods of improving patient access to care are on the rise, such as the advances in Artificial Intelligence (AI) as applied to image based diagnostic fields of medicine such as radiology and pathology. These innovations have begun to be incorporated into global health care strategic planning. For instance, USAID has identified AI as a tool to leap-frog traditional diagnostics, bypassing the need for microscopy as the primary screening mechanism for each slide. Instead, they have proposed that AI screens of digitized slides to identify areas of interest for clinician review, or even infer diagnoses directly using machine learning and pattern recognition (55).

One gap that remains in SSA is limited pathology laboratory participation in External Quality Assurance (EQA) programs for whole slide imaging of histology and cytopathology samples. EQA programs in Africa for epidemic-prone diseases (enteric diseases, meningitis, plague, tuberculosis, and malaria) and blood samples are robust but oncologic EQA lags behind that in HIC (56). EQA providers, such as the College of American Pathologist have put forth validation guidelines for whole slide imaging systems for diagnostic purposes (57). However, the feasibility of implementing such extensive EQA schemes in SSA for cancer care, where laboratories can be tested against their peers is currently limited as they are associated with high costs and require broad collaboration throughout the region. Proficieny testing programs, where external referees send test samples for identification and provide feedback, would be an appropriate benchmark measurement for SSA pathologists that would help identify gaps in cancer training and likely improve diagnostic accuracy.

#### Research and research capacity development

Cancer research output and cancer control programs developed from HICs are not uniformly globally generalizable. Given the unique context, including differences in environmental exposures, oncogenic pathogens, and social, political, and economic pressures, distribution of cancer types, and, indeed cancer biology, may be distinct. Collaborative cancer research programs mindful of local priorities and needs in (and *within*) SSA are critically important for the development of robust cancer registries (58), disease surveillance programs and national preventative care strategies. To this end, telepathology can serve as a centerpiece for cancer research programs in LMICs and as a platform for research mentorship.

Research programs in LMICs have the unique ability to improve health outcomes while also empowering the next generation of local researchers and strengthening research capacity (59). The University of North Carolina Project Malawi Cancer Program uses telepathology to collaborate between physicians and researchers in Malawi and the United States (29). The program was originally developed to accurately diagnose and classify hematologic cancers for patient enrollment into an ongoing observational Kamuzu Central Hospital (KCH) Lymphoma Study, as reported previously. In addition to care coordination and patient follow-up, the weekly telepathology-based meetings, modeled after multi-disciplinary tumor boards, serve to ensure diagnostic accuracy and identify cases and case series for further investigation (50).

Over the course of the KCH Lymphoma Study, initiated in 2013, the format of telepathology research conferences has evolved with changes in staffing and infrastructure. Originally, glass slides scanned in Malawi were presented by US-based pathologists through a Virtual Private Network connection to support primary diagnosis, and aid in interpretation of newly deployed immunohistochemistry. With additional research funding and expansion of diagnostic services, additional Malawian pathologists with advanced training and expertise were employed through government and university support. Telepathology conference “leadership” was shifted to Malawian pathologists who now are the primary drivers of the scanned images shared *via* a readily available video platform. US-based pathologists serve as consultants and second reviewers for study enrollment purposes (50). Of critical importance, is that tissue blocks of enrolled patients are shipped to UNC on a quarterly basis for quality control (QC) and improvement (QI) initiatives and further studies not currently available in Malawi. This form of central review has identified a high concordance rate as well as identified gaps that have led to improvements in Malawi laboratory workflow. Such QC/QI initiatives are important for quality research programs, clinical care, and laboratory accreditation.

In addition to the primary aims of the KCH Lymphoma Study which rely on accurate classification, observations of clinically unique cases or case series have emerged from the telepathology program. These have, in turn, served as primary output for Malawian early-career cancer investigators who aim to develop independent research careers and successfully compete for independent funding. The pathologists themselves are now heavily involved in programmatic development, research output, and local cancer registry leadership.

Other telepathology programs in other LMICs, have developed models based around collaborative telepathology systems. A recent successful example is the collaboration between The City Cancer Challenge Foundation (C/Can) and the American Society for Clinical Pathology in their work establishing a telepathology platform in Yangon, Myanmar (60). The program was based off an extensive needs-assessment at the local level and development of a site-specific intervention with an in-depth analysis of intervention outcome that followed. It serves as an excellent model of project development and deployment.

As noted above, there are challenges and obstacles to research-based telepathology programs. While the questions being addressed are mindful of local need, programs rely on grant funding and investigator time/availability. Often, the time from grant application to grant funding and necessary regulatory approval is prolonged. Moreover, sustainability and growth requires sufficient research output and broadening of programmatic goals for additional grant application, and diversification of funding sources (29), including local governmental support and engagement.

Telepathology can be a critical service when establishing research programs in an LMIC. It provides the infrastructure from which improved training, data collection, and research capacity can take place, all while ensuring local ownership of the research (61).

## Conclusions

There is inadequate access to tissue-based diagnostics in Africa. To ameliorate the dire need for interventions, the solutions to delivering equitable healthcare must be equally as multidimensional and will necessitate a unified collaborative approach to ensure success. Although telepathology has ushered in a groundbreaking means of improving access, collaborating with global colleagues and developing research programs, it cannot comprehensively address dire gaps in caner care delivery. Innovative and collaborative solutions and continued advocacy for global diagnostics remain critically important.

## Data availability statement

The original contributions presented in the study are included in the article/supplementary material, further inquiries can be directed to the corresponding author.

## Author contributions

DR, KP, TT, and YF were responsible for developing, writing, and reviewing the manuscript. All authors contributed to the article and approved the submitted version.

## Funding

This work was supported by the National Institutes of Health/National Cancer Institute D43 CA260641 (YF, PI) and National Institutes of Health/National Cancer Institute U54 CA254564 (YF, MPI).

## Conflict of interest

The authors declare that the research was conducted in the absence of any commercial or financial relationships that could be construed as a potential conflict of interest.

## Publisher's note

All claims expressed in this article are solely those of the authors and do not necessarily represent those of their affiliated organizations, or those of the publisher, the editors and the reviewers. Any product that may be evaluated in this article, or claim that may be made by its manufacturer, is not guaranteed or endorsed by the publisher.
